# Speech Graphs Provide a Quantitative Measure of Thought Disorder in Psychosis

**DOI:** 10.1371/journal.pone.0034928

**Published:** 2012-04-09

**Authors:** Natalia B. Mota, Nivaldo A. P. Vasconcelos, Nathalia Lemos, Ana C. Pieretti, Osame Kinouchi, Guillermo A. Cecchi, Mauro Copelli, Sidarta Ribeiro

**Affiliations:** 1 Brain Institute, Federal University of Rio Grande do Norte, Natal, Brazil; 2 Hospital Onofre Lopes, Federal University of Rio Grande do Norte, Natal, Brazil; 3 Edmond and Lily Safra International Institute of Neuroscience of Natal, Natal, Brazil; 4 Faculdade Natalense para o Desenvolvimento do Rio Grande do Norte, Natal, Brazil; 5 Department of Systems and Computation, Federal University of Campina Grande, Campina Grande, Brazil; 6 Department of Physics, Universidade de São Paulo, Ribeirão Preto, Brazil; 7 Biometaphorical Computing, Computational Biology Center, IBM Research Division, IBM T. J. Watson Research Center, Yorktown Heights, New York, United States of America; 8 Department of Physics, Federal University of Pernambuco, Recife, Brazil; Universitat Pompeu Fabra, Spain

## Abstract

**Background:**

Psychosis has various causes, including mania and schizophrenia. Since the differential diagnosis of psychosis is exclusively based on subjective assessments of oral interviews with patients, an objective quantification of the speech disturbances that characterize mania and schizophrenia is in order. In principle, such quantification could be achieved by the analysis of speech graphs. A graph represents a network with nodes connected by edges; in speech graphs, nodes correspond to words and edges correspond to semantic and grammatical relationships.

**Methodology/Principal Findings:**

To quantify speech differences related to psychosis, interviews with schizophrenics, manics and normal subjects were recorded and represented as graphs. Manics scored significantly higher than schizophrenics in ten graph measures. Psychopathological symptoms such as logorrhea, poor speech, and flight of thoughts were grasped by the analysis even when verbosity differences were discounted. Binary classifiers based on speech graph measures sorted schizophrenics from manics with up to 93.8% of sensitivity and 93.7% of specificity. In contrast, sorting based on the scores of two standard psychiatric scales (BPRS and PANSS) reached only 62.5% of sensitivity and specificity.

**Conclusions/Significance:**

The results demonstrate that alterations of the thought process manifested in the speech of psychotic patients can be objectively measured using graph-theoretical tools, developed to capture specific features of the normal and dysfunctional flow of thought, such as divergence and recurrence. The quantitative analysis of speech graphs is not redundant with standard psychometric scales but rather complementary, as it yields a very accurate sorting of schizophrenics and manics. Overall, the results point to automated psychiatric diagnosis based not on what is said, but on how it is said.

## Introduction

Psychosis is a broad phenomenon that can arise from pathologies such as schizophrenia or mania [1], [2]. Different thought disorders present on these conditions are manifested by disturbances in the normal structure of language. The differential diagnosis of psychosis depends on specific speech disturbances that at present can only be detected by well-trained examiners [3]. Indeed, for over a century the psychiatric interview has been the main tool for mental disease diagnosis [3]. Symptoms are detected by the qualitative analysis of body and verbal language employed to report on everyday facts. Despite the progress achieved by the successive editions of the Diagnostic and Statistical Manual of Mental Disorders, critics remain skeptical about the method's objectivity for differential diagnosis [4]. This contentious background begs a fundamental question for the understanding, diagnosis and treatment of psychosis: is it possible to objectively quantify the disruption in the normal process of thought, and identify precisely the patterns of disruption?

A solution to this problem may come from quantitative speech analysis, using language as a privileged measuring lens into thought. Different aspects of non-pathological language have been studied using complex network models derived from graph theory [5], [6], [7], [8]. A graph represents a network with nodes connected by edges [9], [10]; in the case of language, nodes correspond to words and edges correspond to semantic and grammatical relationships [5], [8]. Formally, graphs are networks defined by G = (N, E) where N = {w_1_, w_2_, w_3_, …} is the set of nodes and E = {(w_i_,w_j_)} is the set of edges between words w_i_ in N and w_j_ in N. Speech graphs belong to the general class of ‘co-ocurrence graphs’, which models co-occurrence patterns between words successively uttered [8]. This means that speech is a directed network, characterized by having each node connected to an ensuing node by a directed edge, indicated by an arrow. Speech also corresponds to a special kind of network called multigraph, in which self-loops (edges connecting a node to itself) and multiple edges (two nodes connected by more than one edge) may occur. Basic measurements for the characterization of those networks can be divided into local measures that describe the neighborhood of a node or the occurrence of sub-graphs (components), and global measures that describe the statistical properties of the entire network [9], [10]. While the interpretation of a graph's meaning depends on what is actually being represented [11], [12], [13], the quantification of its structure may be illuminating. Here we used graphs to quantify structural speech differences between psychotic and normal subjects.

## Results

Oral interviews were recorded with 24 adult subjects, comprising 8 schizophrenic patients, 8 manic patients, and 8 controls without diagnosed mental disorders (Tables S1, S2). As detailed in Methods, we began by applying a standard protocol to certify the psychiatric diagnosis previously given by first response psychiatrists at two public hospitals (SCID). Next, we applied two psychometric scales (PANSS and BPRS) to quantify symptoms at the time of the interview, including psychosis. Then, subjects were asked to report exclusively on a recent dream. Deviations from this anchor topic to report on waking events were used to evaluate “flight of thoughts”, a typical manic symptom [1].

**Figure 1:**
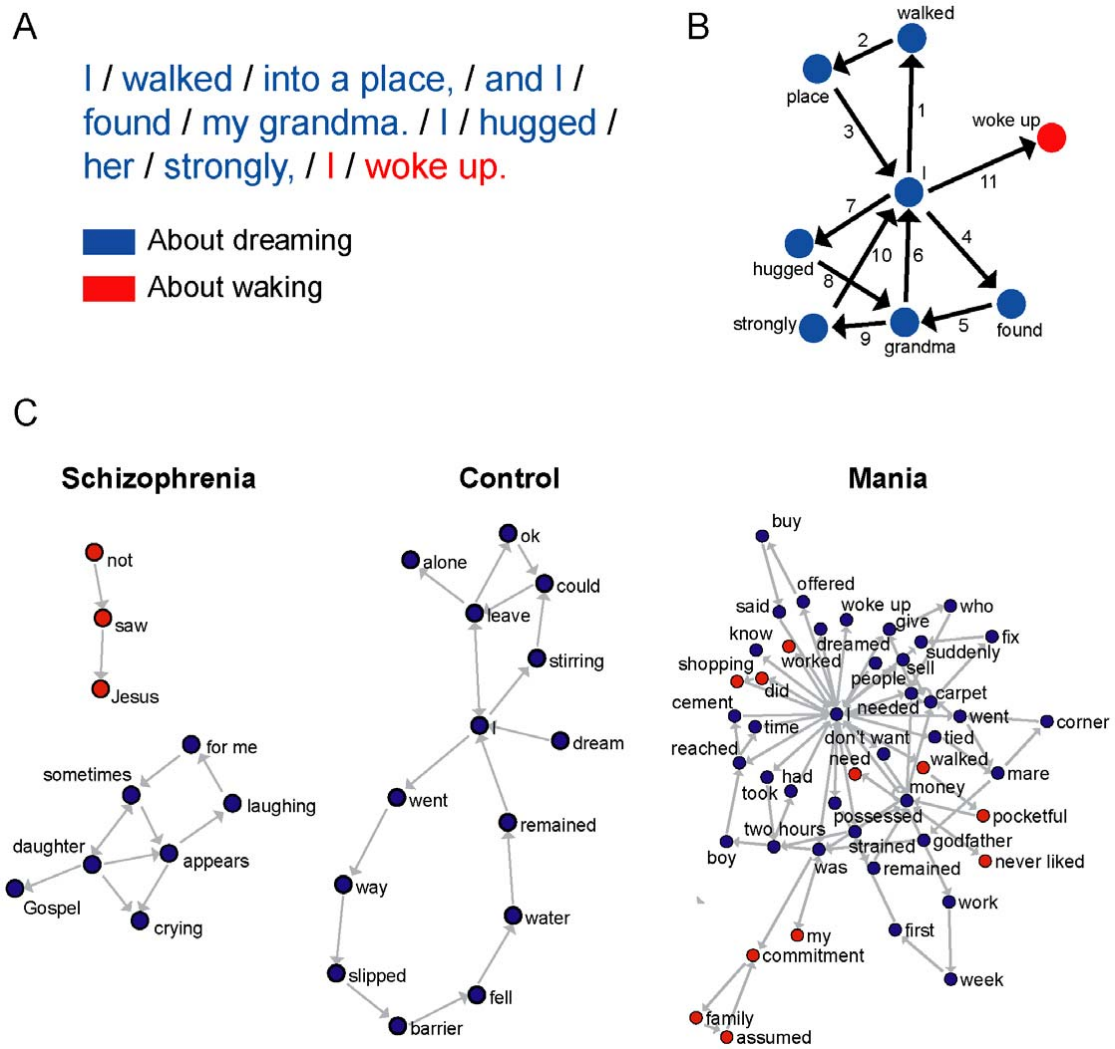
Speech graph analysis in schizophrenia, mania and control reports. A) Subjects were asked to report a recent dream. Each report was transcribed and parsed into canonical grammatical elements (words translated from Portuguese, elements separated by slashes). Parts related to dreaming (blue) were sorted from parts related to waking (red), which were considered deviations from the anchor topic. B) Speech graph from the example shown in A), with edges sequentially numbered. The node “I” appears 3 times in the dream sub-graph (“I walked”, “I found”, “I hugged”), and then once in the waking sub-graph (“I woke up”). C) Speech graph examples representative of the schizophrenics (subject MG), manics (subject AB) and controls (subject OR). Graphs plotted using global energy minimum (GEM). The complete database is available as Supporting Information.

The reports were parsed into backbone speech elements that corresponded to subject, verb and object (Fig. 1A). Each report was represented by a directed multigraph in which each node corresponded to a canonical element (lexeme) and the temporal link between two elements was represented by an edge (Fig. 1B). Elements related to dreaming were sorted from elements related to waking (Fig. 1B). Representative graphs illustrate the major speech differences among schizophrenics, manics and controls, such as amounts of nodes and edges, or recurrence and deviation from the anchor topic (Fig. 1C).

**Figure 2:**
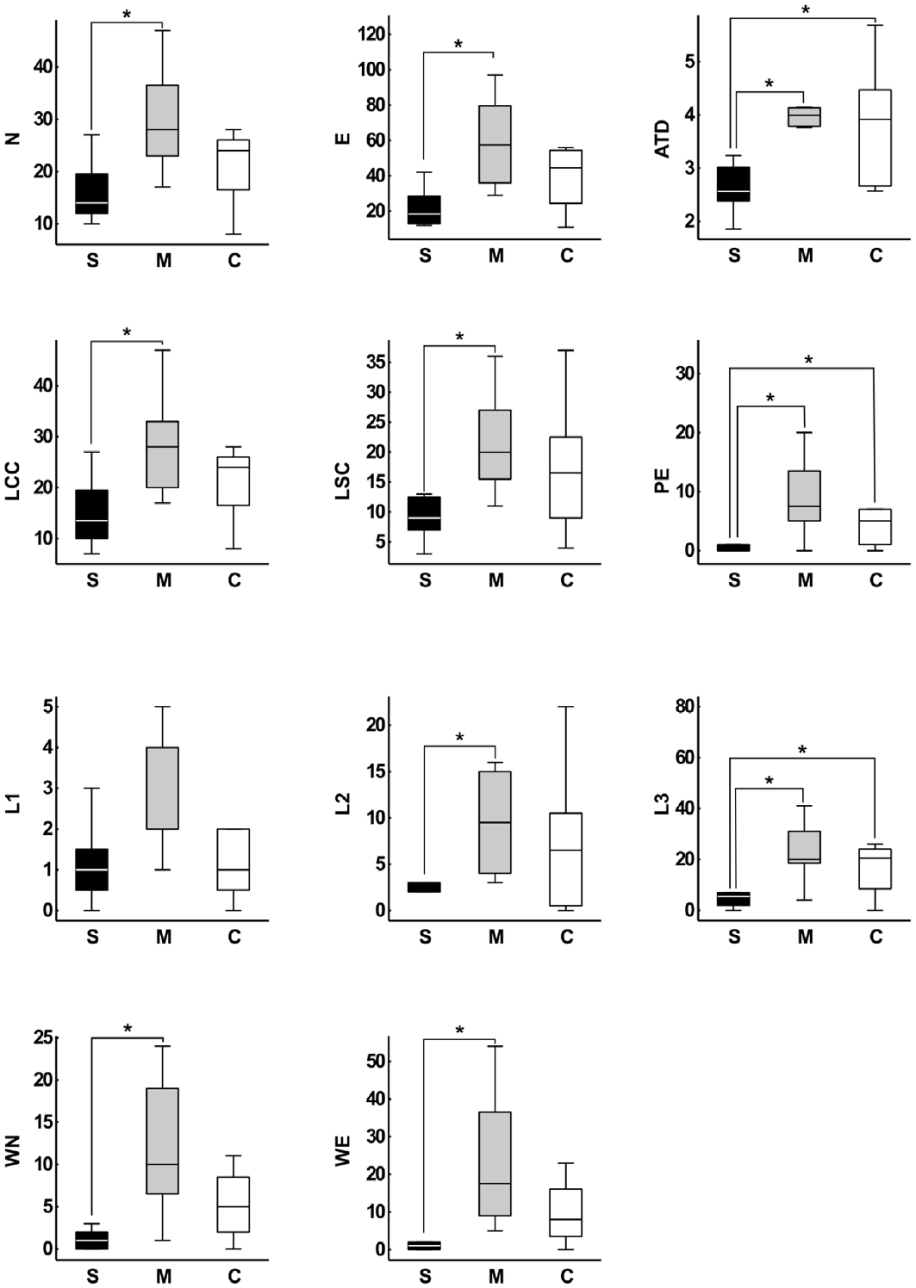
Local speech graph measures are significantly different for schizophrenics (S), manics (M) and controls (C). Boxplots of speech graph measures. General measures N (*P = *0.0028) and E (*P = *0.003). Connectivity-related measures LCC (*P = *0.005), LSC (*P = *0.0078) and ATD (*P = *0.007 and *P = *0.016). Recurrence-related measures PE (*P = *0.0031 and *P* = 0.0143), L2 (*P* = 0.0025) and L3 (*P = *0.005 and *P* = 0.0160). Waking-related measures WN (*P = *0.0059) and WE (*P = *0.0014). Asterisks indicate statistical significance with Bonferroni correction. All the individual raw data and complete statistical results are presented in Tables S3 and S4, respectively.

**Figure 3:**
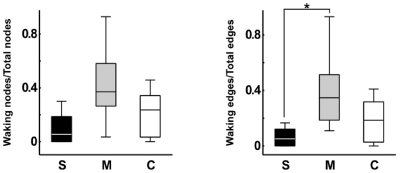
In comparison with schizophrenics, manic patients had significantly more flight of thoughts (higher rate of dream report interruptions to comment on waking events). Ratios of waking nodes/total nodes and waking edges/total edges across groups. For edges, manics scored significantly higher in this ratio than schizophrenics (Kruskal-Wallis *P = *0.0397, Wilcoxon Ranksum test *P = *0.0146). A non-significant but similar trend was observed for nodes (Kruskal-Wallis *P = *0.0597, Wilcoxon Ranksum test *P = *0.0258). Asterisk indicates statistical significance with Bonferroni correction.

**Figure 4:**
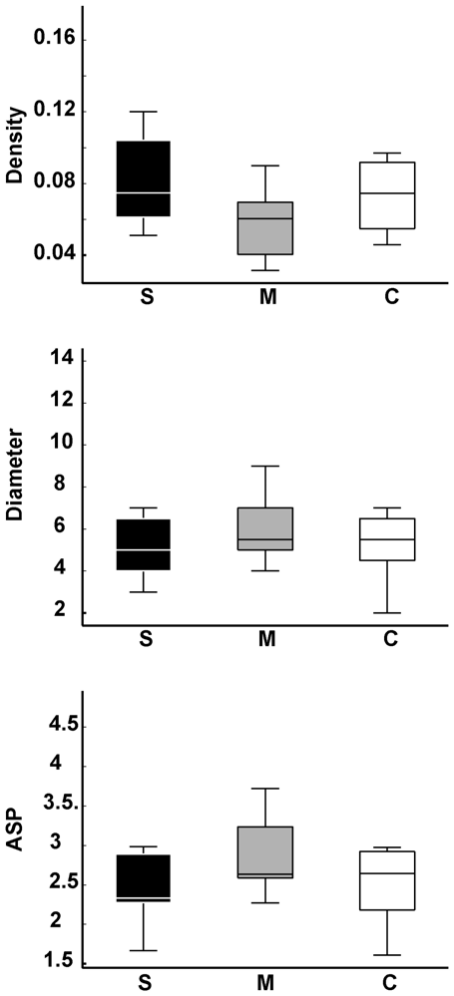
Global speech graph measures were not significantly different for the different groups. Using the raw data, medians and variances were very similar across groups.

To quantify these effects, we began by calculating eleven local measures according to the following categories: General (Fig. 1), including number of nodes (N) and number of edges (E); connectivity-related (Fig. S1), including number of nodes on the largest connected component (LCC), number of nodes on the largest strongly connected component (LSC), and average total degree (ATD); recurrence-related (Fig. S1), including number of parallel edges (PE), and number of loops with one, two or three nodes (L1, L2, L3); and topic deviation (Fig. 1), including number of waking nodes (WN) and waking edges (WE). Manics scored significantly higher than schizophrenics in nearly all measures (Fig. 2 and Tables S3, S4). Manic reports displayed more nodes and edges than reports from schizophrenics, reflecting the increase in the amount of talking that defines “logorrhea”. Manics also scored significantly higher for measures related to connectivity and recurrence than schizophrenics, which points to the impoverished speech of the latter. Finally, manic reports contained significantly more waking nodes and edges than schizophrenic reports, revealing “flight of thoughts”. This was confirmed by calculating ratios of waking nodes/total nodes and waking edges/total edges across groups (Fig. 3). We also calculated three global measures for the statistical analysis of the connectivity structure of the entire network: Density (D), diameter (DI) and average shortest path (ASP); no significant group differences were obtained (Fig. 4).

**Figure 5:**
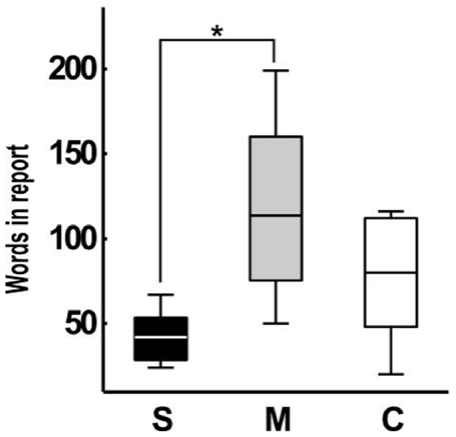
Schizophrenics produced significantly less words per report than manics. Boxplots of total number of words per report across groups. (Kruskal-Wallis test across groups, *P = *0.0067; Wilcoxon Rank Sum test between schizophrenics and manics *P = *0.0006; between manics and controls *P = *0.2911, and between schizophrenics and controls *P = *0.0650). Asterisk indicates statistical significance with Bonferroni correction.

**Figure 6:**
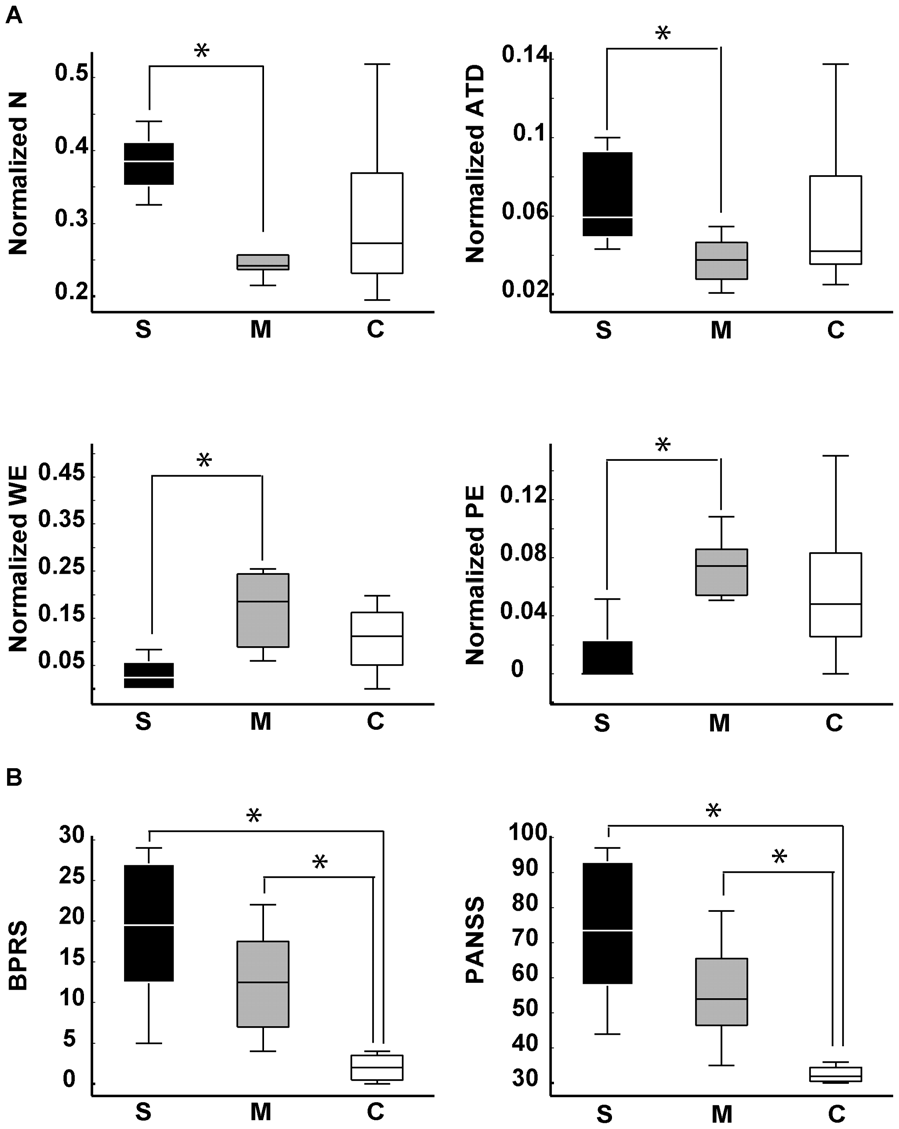
Schizophrenics and manics are better distinguished by normalized speech graph measures than by psychometric scales BPRS and PANSS. A) Boxplots of normalized data show differences between schizophrenics and manics for N (*P = *0.0104), ATD (*P = *0.0051), PE (*P = *0.0044), and WE (*P = *0.0146). B) Boxplots of BPRS and PANSS scores show significant differences between psychotic patients and controls (S>C with *P = *0.0003, M>C with *P = *0.0012; for PANSS: S>C with *P = *0.0003, M>C with *P = *0.0006). No significant differences were found between schizophrenics and manics (BPRS S>M with *P = *0.1377; PANSS S>M with *P = *0.1108). Asterisks indicate statistical significance with Bonferroni correction. All the individual normalized data and complete statistical results are presented in Tables S3 and S4, respectively.

Since schizophrenics spoke significantly less per report than manics (*P = *0.0006; Fig. 5), their several differences at the level of local graph measures could potentially be explained by this major verbosity difference. Yet, we found four speech graph local measures to be significantly different between groups even when differences in the number of words per report were discounted (Fig. 6A, Fig. S2, Table S4): Graphs from the schizophrenic reports presented more nodes per word (*P = *0.0104) and a higher average total degree per word (*P = *0.0051) than reports from manics. Furthermore, graphs from the manic group still displayed more waking edges (*P = *0.0146) and more parallel edges (*P = *0.0044) than graphs from the schizophrenic group. Thus, manics and schizophrenics showed markedly different tendencies to reiterate or abandon a conversation topic, even when the data were normalized by the number of words per report. In comparison, no significant differences were detected between schizophrenics and manics with regard to BPRS and PANSS scores, two standard scales for the quantification of psychotic symptoms (Fig. 6B).

**Figure 7:**
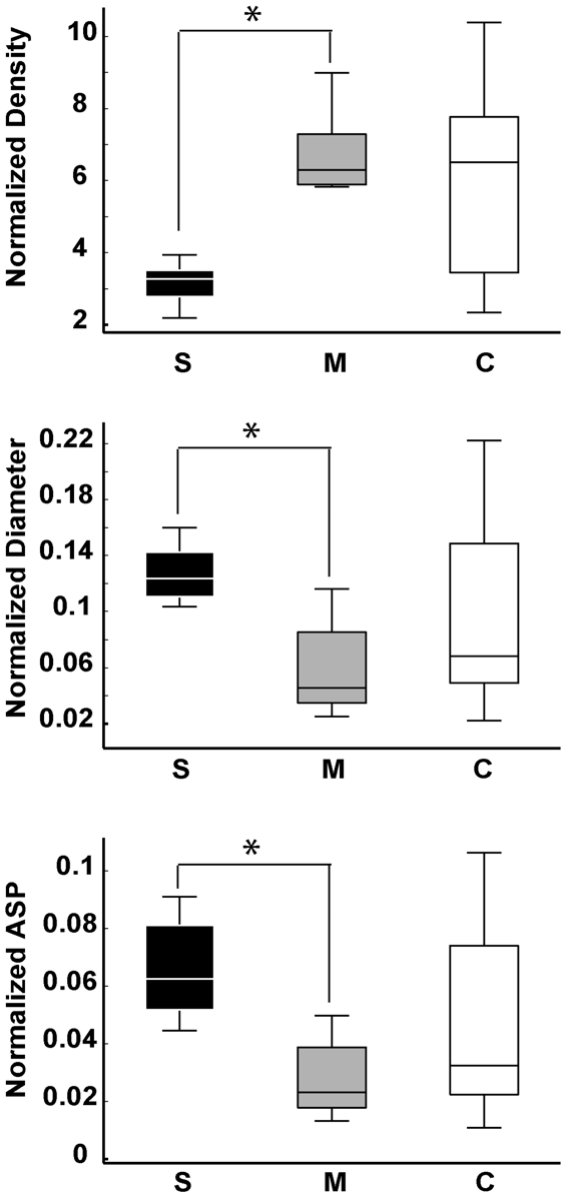
Normalized global speech graph measures were significantly different between schizophrenics (S) and manics (M). Boxplots of normalized data show significant differences between schizophrenics and manics for Density (*P* = 0.007), Diameter (*P* = 0.0138) and ASP (*P* = 0.0104) with small variance for both groups. In contrast, control subjects yielded a wide span of values between manics and schizophrenics, with high variance.

While data normalization by the number of words per report decreased the number of significant group differences for local graph measures, it had the opposite effect with regard to global measures. As shown in Figure 7, speech graphs from schizophrenics and manics showed major differences in normalized global network properties, and were easily separated by each of the three measures due to the reduced inter-individual variance within each pathological group. Manics produced significantly denser graphs than schizophrenics (*P = *0.007), with significantly smaller diameter (*P = *0.0138) and average shortest path (*P = *0.0104) in the former than in the latter. Interestingly, controls yielded intermediate levels between manics and schizophrenics, spanning a wide range of values that reflect the increased inter-individual differences among non-pathological subjects.

**Figure 8:**
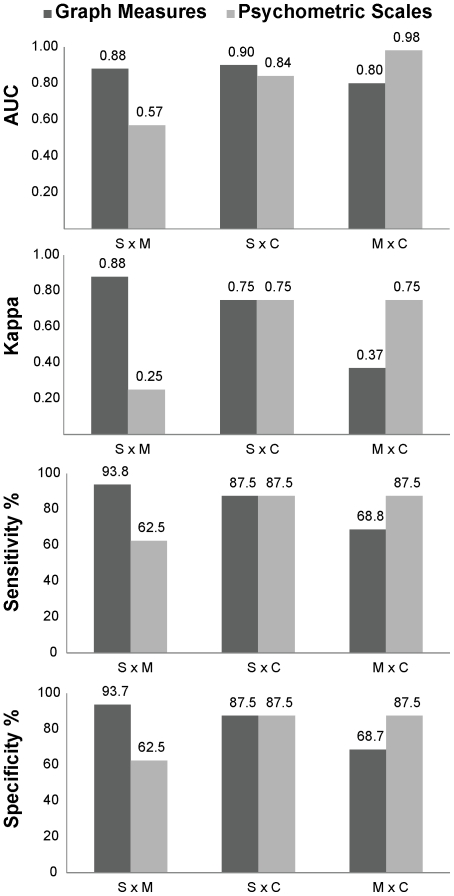
Speech graph measures provide better differential diagnosis of mania and schizophrenia than standard psychometric scales (BPRS and PANSS). Group sorting using graph measures as inputs to the NB classifier was excellent to separate schizophrenics from manics. The measures used as inputs were N, E and ATD for S x M; N, L1 and L2 for S x C; L1, L2 and L3 for M x C. In contrast, group sorting using BPRS and PANSS total scores as inputs for the classifier was successful in separating controls from psychotic patients (either S or M), but sorting between schizophrenics versus manics (S x M) was poor.

To investigate the feasibility of automated differential diagnosis based on speech graph analysis, we trained a naive Bayes (NB) classifier with different subsets of graph measures as inputs. The data were normalized by the number of words in each report, in order to discount the effects of normal inter-individual verbosity differences. Furthermore, the inputs were restricted to data that could be obtained without having to resort to an interpretation of the meaning of the reports, i.e. waking nodes and edges were not employed. Sensitivity, specificity, the area under the receiver operating characteristic curve (AUC) [14] and the kappa statistic [15] were used as metrics of classification quality. Our approach objectively and accurately distinguished schizophrenic from manic reports (Fig. 8), and was comparable to the inter-rater reliability of SCID for the distinction between schizophrenics and controls, but not for the distinction between manics and controls [16], [17]. In contrast, when the scores from the psychometric scales BPRS and PANSS were used as inputs to the classifier, it was possible to distinguish controls from psychotic patients, but not schizophrenics from manics (Fig. 8). Indeed, none of the graph measures correlated significantly with BPRS and PANSS scores (Table S5).

To further investigate the issue of classifier accuracy, we compared the group classification obtained by the NB model [18], [19] with four other binary classifiers: Radial Basis Function (RBF), Multi-Layer Perceptron (MLP), Support Vector Machine (SVM), and Decision Tree (DT). As shown in Table S6, all classifiers sorted manics from schizophrenics better when the inputs were speech graph measures, in comparison with psychometric data. The best results were obtained using RBF and NB (93.8% of sensitivity and 93.7% specificity). The sorting of schizophrenics versus controls was similar for psychometric and speech graph measures, but the sorting of manics versus controls was better when psychometric measures were used as inputs (Table S6).

## Discussion

The results show for the first time that a graph analysis of the speech produced by psychotic patients can be used to quantitatively sort manics from schizophrenics. Indeed, this approach allowed for a very accurate discrimination of the pathological groups of interest using various binary classifiers, reaching more than 93% of sensitivity and specificity in the separation of schizophrenics from manics. In contrast, sorting based on the scores of two standard psychiatric scales (BPRS and PANSS) reached only 62.5% of sensitivity and specificity. This indicates that the quantitative analysis of speech graphs is not redundant with the major psychometric scales but rather complementary, because it measures speech structure symptoms not well grasped by those instruments.

Our approach was not purely topological, since two out of the fourteen graph measures investigated in the present study required semantic node labeling (i.e., waking versus dreaming). Notwithstanding, none of the remaining measures required any interpretation beyond the differentiation of lexemes, strictly at the grammatical level. Importantly, the data fed to the binary classifiers did not include those two waking-related measures.

Symptoms such as poor speech, logorrhea and flight of thoughts were detected by graph analysis even when inter-individual differences in verbosity were accounted for. Manics produced more parallel edges per word and more waking edges per word than schizophrenics. This means that the “logorrhea” typical of manics [1] comes not only from the excess of words, but from a discourse that branches more and returns more times to the same topic, in comparison with schizophrenic group. Likewise, “flight of thoughts” cannot be trivially explained by increased verbosity, but rather corresponds to a structural feature of manic speech. On the other hand, schizophrenics displayed more nodes per word and a higher average total degree per word than manics. This means that schizophrenics tended to address topics only once, neither branching nor recurring, a reflection of the “poor speech” typical of these patients [1].

It has been recently observed that the amount of loops in a network is inversely correlated with its dynamical stability [20]. In our study, the presence or absence of loops is directly related to the recurrence, or lack thereof, of similar thoughts in the course of the interviews. The fact that mania reports have more parallel edges per word than reports from schizophrenics may therefore reflect the decreased stability of manic speech. On the other hand, the increase in schizophrenics of the amount per word of nodes and average total degree points to the increased stability of schizophrenic speech, in comparison with manic speech. These features likely influence disease course, producing cyclic symptom changes in manics [3] and persistent symptoms with monotonic clinical evolution in schizophrenics [3].

Manics produced significantly denser graphs than schizophrenics, with significantly smaller diameter and average shortest path. Small variance characterized both psychotic groups, while controls yielded a wide range of values with high variance. These results reveal the strong pathological determination of the global network measures, which seems to constrain the structure of manic and schizophrenic speech in opposite ways. In contrast, controls free from such a determination expressed the global features of speech with much larger inter-individual differences, suggesting that the structural variance of speech increases in the absence of pathological constraints.

Our results connect the quantification of mental disorders with research on computational semantic analysis, fueled by the expanding availability of online text corpora and computational resources [21], [22]. The data demonstrate that the alteration of the thought process manifested in the speech of psychotic patients can be objectively measured using graph-theoretical tools, developed to capture analytically some intuitive features of the normal and dysfunctional flow of thought, such as divergence and recurrence. The classification accuracy obtained using these graph features provides validation to the method, as it matches the consensus of experts. By the same token, the results indicate that the differential diagnosis of psychosis can be greatly improved by speech graph analysis. The networks studied here were relatively small, reflecting the difficulties in obtaining speech graphs from psychotic patients interviewed during clinical examinations. Future work should challenge the robustness of our results, assessing their clinical significance on substantially larger samples. We propose that such a quantitative approach may soon allow doctors to identify mental disorders and track the progress of treatment in an automated manner [23], i.e. through a psychiatric Turing test [24].

## Materials and Methods

### Subjects

Study approved by the Research Ethics Committee of the Federal University of Rio Grande do Norte (permit #102/06-98244). As stated by the procedure specifically approved by the Ethics Committee, written informed consent was obtained from all subjects after they were read a document with detailed information about the nature and possible consequences of the study, had verbally discussed any possible concerns with the experimenter, and had provided clear indication that they had understood the procedure. During the psychiatric interview, patients were examined for major changes in state and level of consciousness (e.g. drowsiness, torpor), for signs of autopsychic and allopsychic disorientation (e.g. inability to remember name, age, spatial localization), and for signs of reduced mnemonic and cognitive capacity. All psychotic subjects were medicated and out of the acute psychotic phase at the onset of the study, so typically they were in good capacity to provide informed consent. Signs of disorientation or reduced mnemonic capacity were detected in 3 out of 16 psychotic subjects (1 manic and 2 schizophrenics). In the case of these subjects, the experimenter also obtained written informed consent on their behalf from their legal guardians (next of kin). All the signed forms with written informed consent were archived by the corresponding author. Schizophrenic and manic patients were pre-diagnosed independently by first response psychiatrists. All subjects were interviewed and digitally recorded during daytime by a psychiatrist of our team (NBM). Demographic and clinical data are shown on Tables S1 and S2.

### Interviews for Psychiatric Assessment and Anchor Topic

Interviews began with confirmation of the diagnostics established by first-response psychiatrists (“Structured Clinical Interview for DSM-IV”, SCID Portuguese version) [2]. Due to diagnostic mismatch, 5 interviews were discarded and substituted. We also applied the “Positive and Negative Syndrome Scale” (PANSS) [25] and the “Brief Psychiatric Rating Scale” (BPRS) [26] to further quantify psychiatric symptoms. As expected, the scores on these scales were strongly correlated across subjects (Fig. S3). Subjects were then requested to report exclusively on their most recently experienced dream, which served as an anchor topic. Recordings proceeded without interference from the interviewer. Interviews lasted 20–60 minutes, yielding in average 84 words per report related to the anchor topic.

### Speech Graph Measures

Anchor topic reports were blindly transcribed by 2 different researchers. Next, the words were converted to canonical forms (lexemes). The reports were then parsed into grammatical elements corresponding to subject, verb and object, and were then converted to graphs. We calculated eleven local graph measures (see Fig. S1), as follows:

Nodes (N) = Number of elements in N;

Edges (E) = Number of elements in E;

Average Total Degree (ATD) = Mean k_i_ = k _in,i_+k _out,i_, where the in-degree k_in,i_ of the node *i* is defined as the number of edges pointing to *i*; its out-degree k _out,i_ is defined as the number of edges departing from *i*;

Largest Connected Component (LCC) = Total number of nodes comprising the largest sub-graph in which each node is connected to each other one through a path in the sub-graph; the measure applies to the undirected version of the graph;

Largest Strongly Connected Component (LSC) = Total number of nodes comprising the largest sub-graph in which all nodes are mutually reachable, i.e., there is a path from node *a* to node *b*, and there is a path from node *b* to node a; the measure applies to the directed version of the graph;

Parallel Edges (PE) = Total number of edges linking the same pair of nodes more than once;

Loops with 1 Node (L1) = Trace of the adjacency matrix that represents the graph. Same as self-loops;

Loops with 2 Nodes (L2) = Trace of the squared adjacency matrix;

Loops with 3 Nodes (L3) = Trace of the cubed adjacency matrix;

Waking Nodes (WN) = Total number of nodes used to talk about waking events;

Waking Edges (WE) = Total number of edges used to talk about waking events.

We also calculated three global measures of the speech graphs by excluding self-loops and parallels edges and, in the case of DI and ASP, by further transforming the graphs into derived networks without directionality:

Density (D) = E′/N^2^, with E′ = E - (L1+PE);

Diameter (DI) = Length of the longest shortest path between the node pairs of a network;

Average Shortest Path (ASP) = Average length of the shortest path between pairs of nodes of a network;

Overall, we calculated 2 general measures (N and E), 3 connectivity-related measures (LCC, LSC, and ATD), 4 recurrence-related measures (PE, L1, L2, and L3), 2 waking-related measures (WN and WE) and 3 global measures (D, DI and ASP). Since manic reports were significantly wordier than schizophrenic reports (Fig. 5), we compared the raw data to results obtained by normalizing each graph measure by the total number of words in each report. All general and connectivity-related graph measures, as well as the recurrence-related measure PE and two global measures (DI and ASP) were calculated using the Network Analysis Toolkit (http://nwb.cns.iu.edu/). Recurrence-related measures comprising L1, L2, L3 were calculated using Matlab. Measures related to wakefulness (WN and WE) were visually counted. Density was calculated using Excel. Kruskal-Wallis tests followed by Wilcoxon Ranksum tests with Bonferroni correction were used to assess significant differences (corrected α = 0.0166).

### Automated Classification of Speech Graphs

In the case of small datasets such as those investigated here, the Naïve Bayes (NB) classifier has been shown to provide superior performance [27], [28]. The NB classifier can be modeled as a directed acyclic graph, in which all edges go from a single “root” node representing the class to potentially many “children” nodes, representing the attributes [29]. Let X be a vector of random variables representing the attribute values, and C be a random variable representing the class of an instance. Moreover, let x = (x_1_,x_2_,…,x_N_) be a particular value of the attributes and let c be a particular class label. It is then possible to use the Bayes' rule to estimate the probability of each class based on a given attribute value [29]. To that end, each classifier attribute corresponded to a specific speech graph measure (e.g. ATD, LCC, etc) or a given psychometric measurement (PANSS and BPRS). Class labels corresponded to the different groups studied (manic, schizophrenic and control). To address the issue of classifier accuracy, we compared the group classification obtained by the NB model [18], [19] with four other binary classifiers: Radial Basis Function (RBF), Multi-Layer Perceptron (MLP), Support Vector Machine (SVM) and Decision Tree (DT). All the classifiers were implemented using Weka software [30]. A cross-validation procedure was implemented to take full advantage of the sample size. Classifier inputs consisted of graph measures normalized by the number of words per report; classifier outputs were binary decisions in the form “is this graph from a given group or not”. After identifying the measures that best separated the groups in each comparison, the classifier was trained with particular measure combinations for each comparison: For schizophrenics versus manics we used N, E and ATD; for schizophrenics versus controls we used N, L1 and L2; for manics versus controls we used L1, L2 and L3. To quantitatively distinguish reports from schizophrenics, manics and controls, receiver operating characteristic (ROC) curves were built based on the outputs of the classifier [14]. Sensitivity, specificity and the area under the ROC curve (AUC) were used as a metric of classification quality. The ROC curve is a plot of sensitivity (true positive rate) on the y axis, and 1 – specificity (false positive rate) on the x axis. The area under the ROC curve (AUC) reveals the probability that the classifier will assign a higher score to a randomly chosen positive instance than to a randomly chosen negative instance. AUC values around 0.5 mean a random classification, while values above 0.75 indicate good classification. To verify the agreement between the diagnostic classifications obtained with DSM IV criteria and graph measures, we calculated the kappa statistic, an inter-rater agreement measure for which values around 0.6 indicate a good agreement, and values above 0.8 indicate excellent agreement [15].

## Supporting Information

Figure S1 **Examples of speech graph measures calculated in this study.**
(TIF)

Figure S2 **Boxplots of normalized graph attributes whose differences were not statistically significant.** General attribute E; connectivity-related attributes LCC and LSC; recurrence-related attributes L2 and L3; and waking-related attributes WN and WE. Notice that WN and WE, after normalization for the number of words per report, show a non-significant M>S trend. P values in Table S4.
(TIF)

Figure S3 **BPRS and PANSS scores for all subjects (N = 24).** There was a tight correlation between the BPRS and PANSS scores across all groups (schizophrenics R2 = 0.9301, manics R2 = 0.8823, controls R2 = 0.9812).
(TIF)

Table S1 **Socio-demographic characteristics including age (mean age and standard error), sex (absolute number of subjects and percentage), years of education (mean years and standard error), and marital status (absolute number of subjects and percentage).** Psychiatric assessment of psychotic subjects including age of onset (mean age and standard error) and medication used (absolute number of subjects and percentage).
(TIF)

Table S2 **Socio-demographic characteristics and psychiatric assessment of psychotic subjects for all subjects.** Typical anti-psychotics (TAP) included haloperidol, levomepromazin, and clorpromazin. Atypical anti-psychotics (ATAP) included olanzapine, risperidone and quetiapine.
(TIF)

Table S3 **Speech graph attributes (raw data) and psychometric scales BPRS and PANSS.** Subjects indicated by name and surname initials.
(TIF)

Table S4 **P values obtained on the Kruskal-Wallis (KW) test followed by Wilcoxon-Ranksum test with Bonferroni correction for pairwise group comparisons of raw and normalized data for schizophrenics (S), manics (M) and controls (C).** Statistically significant differences indicated in red, near-significant trends indicated in blue.
(TIF)

Table S5 **There were no significant correlations between normalized graph attributes and psychometric scales (BPRS and PANSS scores).** Shown are Rho and P values of Spearman correlations (corrected α = 0.0166).
(TIF)

Table S6 **Classification quality obtained for speech graph and psychometric measures.** Five different binary classifiers were used: Naïve-Bayes (NB), Support Vector Machine (SVM), Decision Tree (DT), Multi-Layer Perceptron (MLP), and Radial Basis Function (RBF).
(TIF)
