# Supplementary figures and images for: Speech Graphs Provide a Quantitative Measure of Thought Disorder in Psychosis
Source: PLoS One. 2012 Apr 9;7(4):e34928. doi: 10.1371/journal.pone.0034928 (PMC3322168; doi:10.1371/journal.pone.0034928)

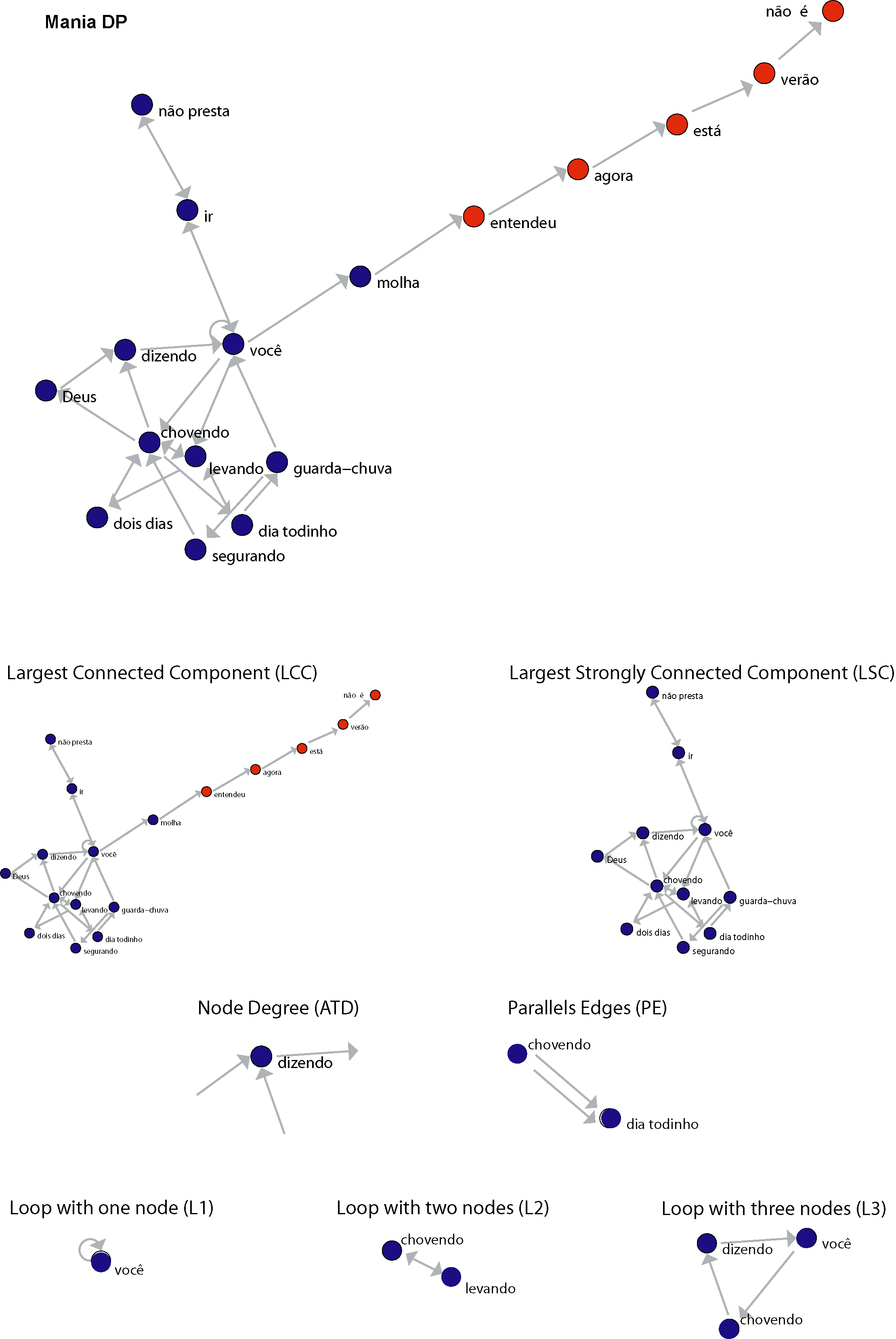

Supplement: Figure S1 — Examples of speech graph measures calculated in this study. (TIF) [file pone.0034928.s001.tif]

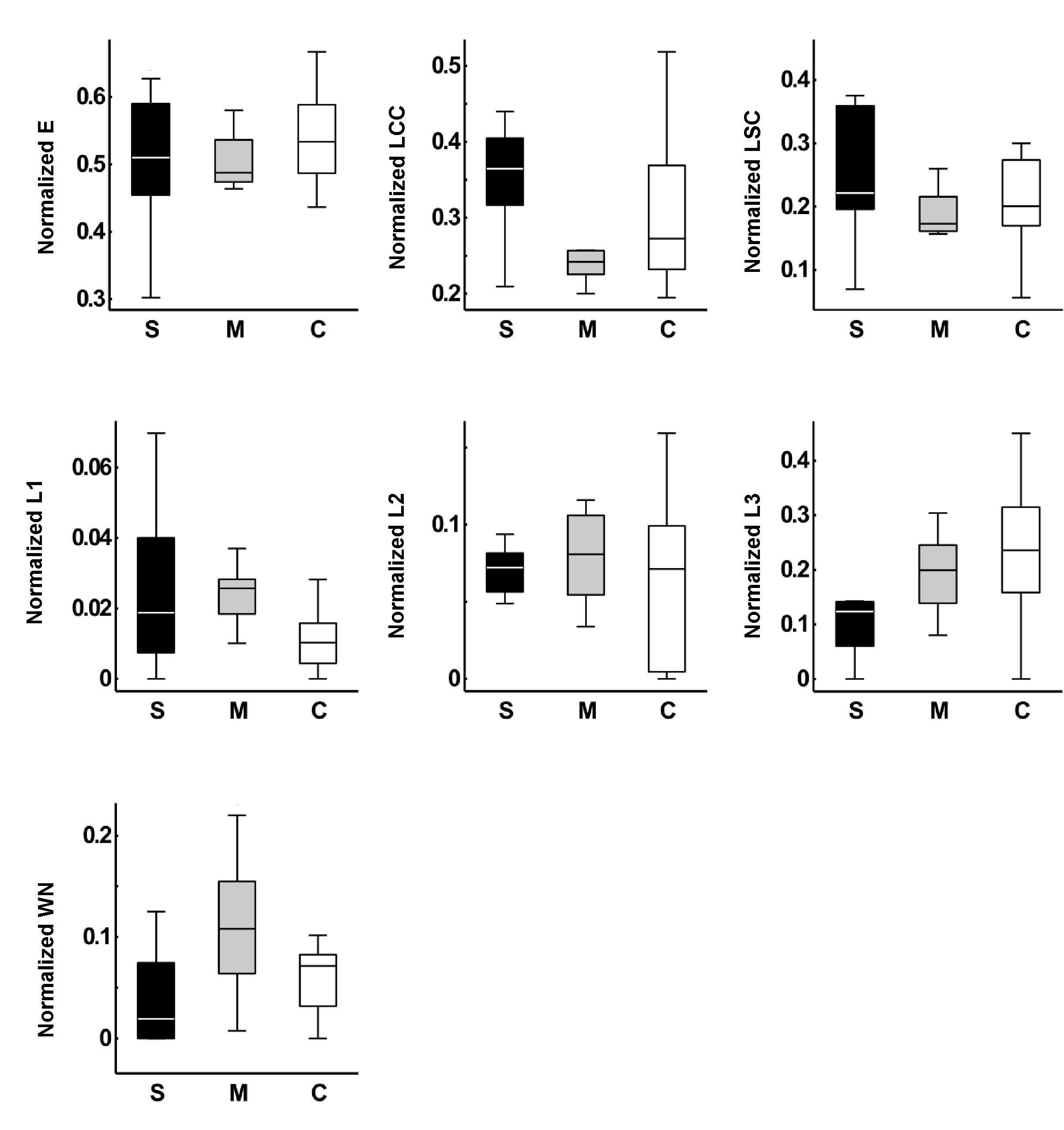

Supplement: Figure S2 — Boxplots of normalized graph attributes whose differences were not statistically significant. General attribute E; connectivity-related attributes LCC and LSC; recurrence-related attributes L2 and L3; and waking-related attributes WN and WE. Notice that WN and WE, after normalization for the number of words per report, show a non-significant M>S trend. P values in Table S4. (TIF) [file pone.0034928.s002.tif]

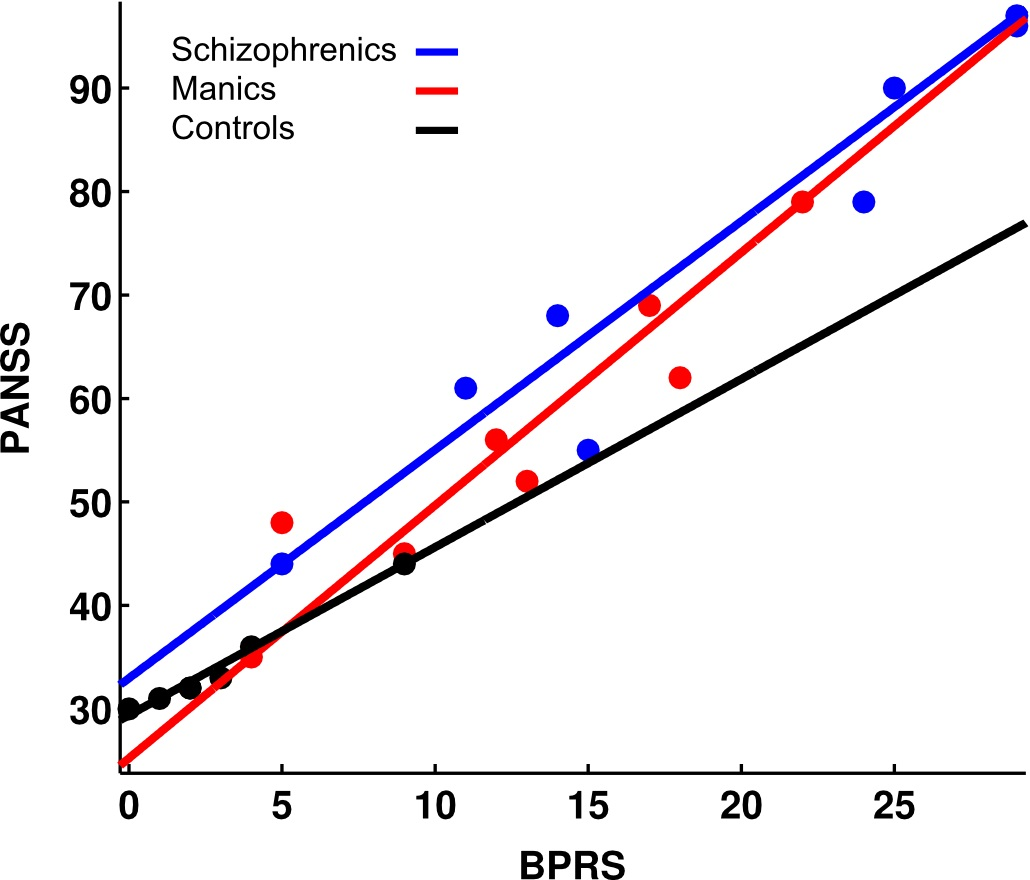

Supplement: Figure S3 — BPRS and PANSS scores for all subjects (N = 24). There was a tight correlation between the BPRS and PANSS scores across all groups (schizophrenics R2 = 0.9301, manics R2 = 0.8823, controls R2 = 0.9812). (TIF) [file pone.0034928.s003.tif]

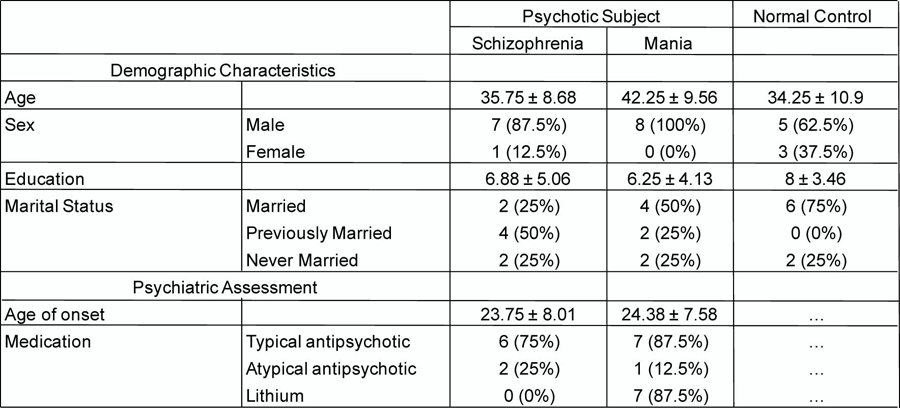

Supplement: Table S1 — Socio-demographic characteristics including age (mean age and standard error), sex (absolute number of subjects and percentage), years of education (mean years and standard error), and marital status (absolute number of subjects and percentage). Psychiatric assessment of psychotic subjects including age of onset (mean age and standard error) and medication used (absolute number of subjects and percentage). (TIF) [file pone.0034928.s004.tif]

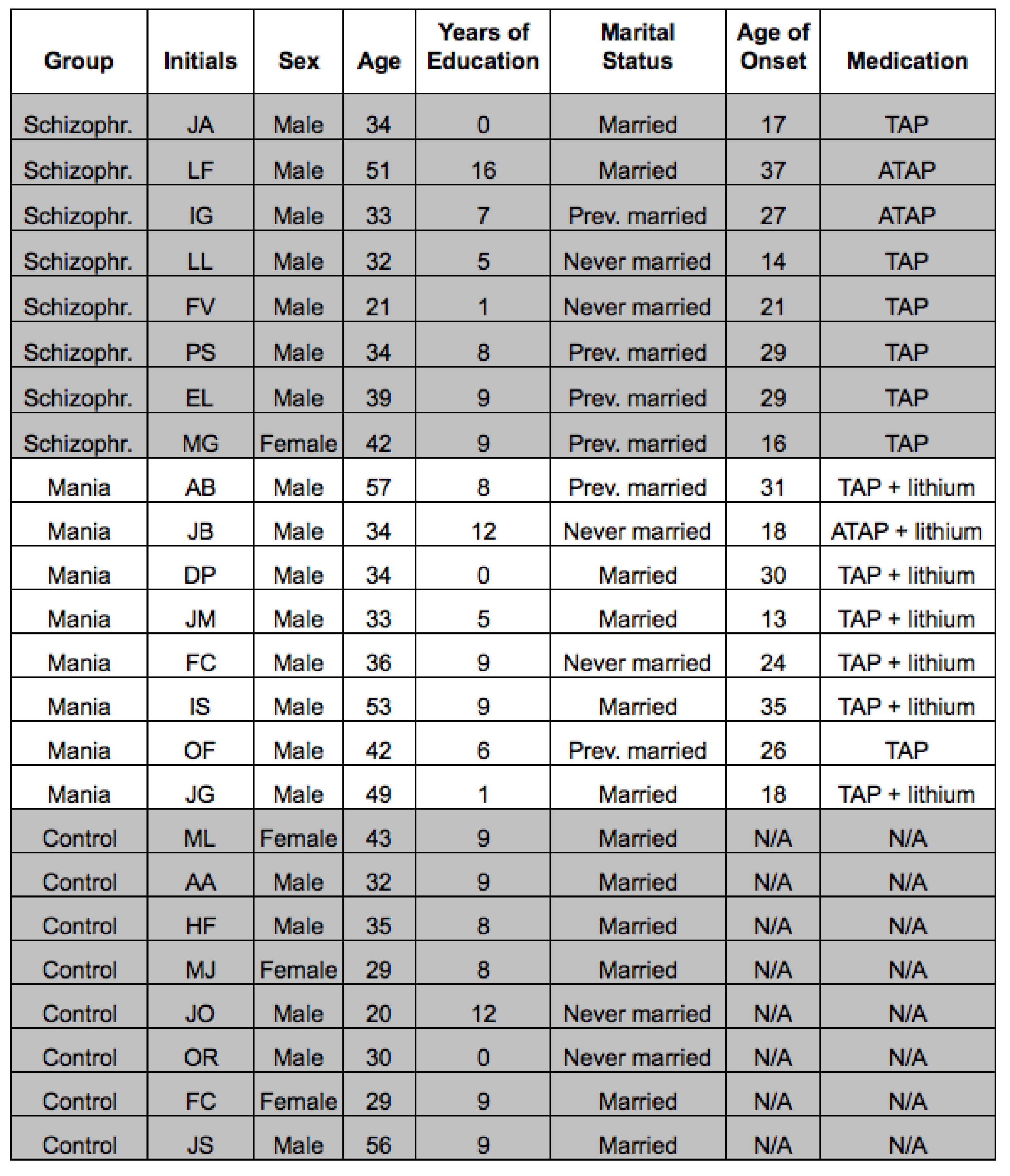

Supplement: Table S2 — Socio-demographic characteristics and psychiatric assessment of psychotic subjects for all subjects. Typical anti-psychotics (TAP) included haloperidol, levomepromazin, and clorpromazin. Atypical anti-psychotics (ATAP) included olanzapine, risperidone and quetiapine. (TIF) [file pone.0034928.s005.tif]

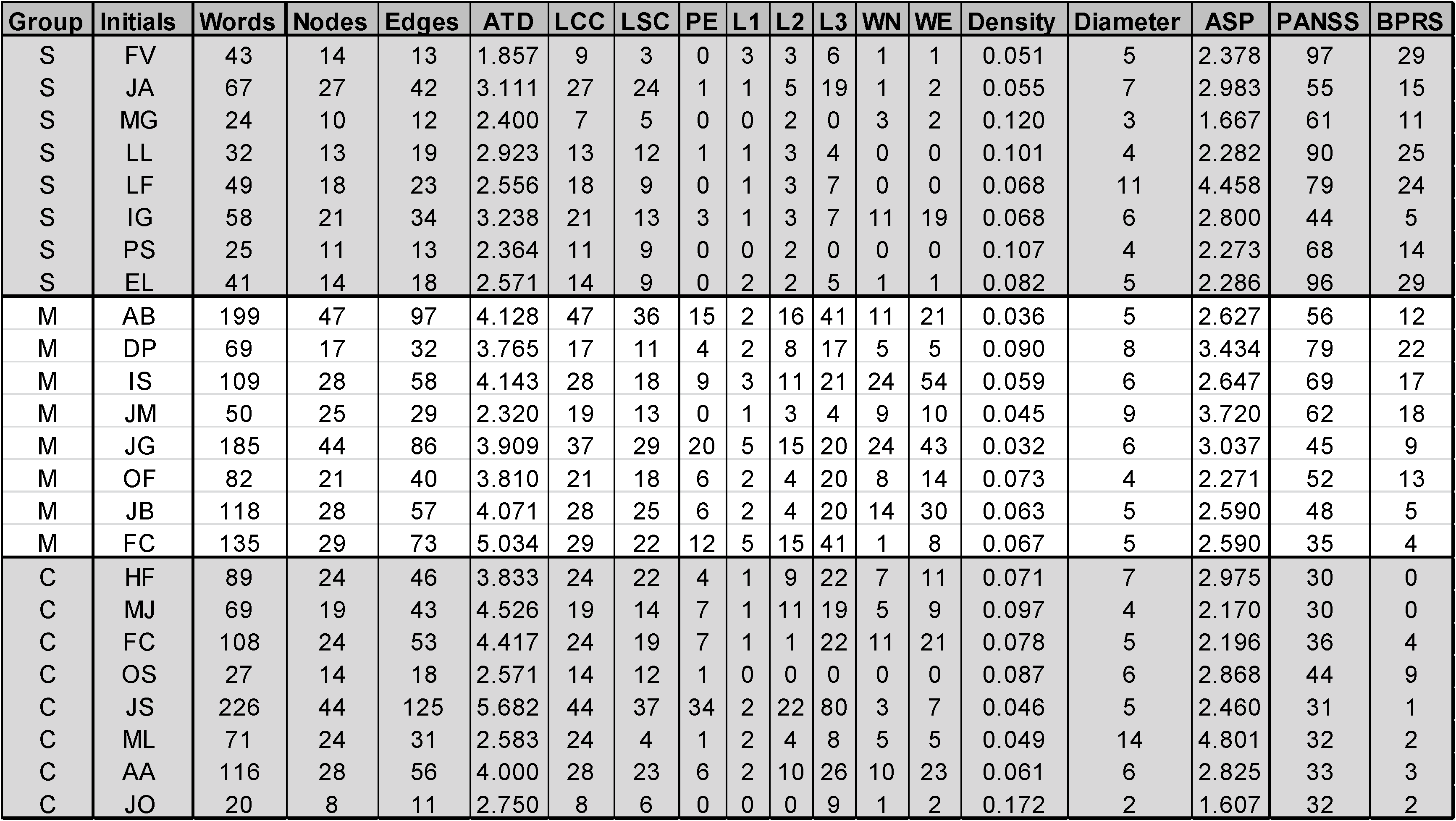

Supplement: Table S3 — Speech graph attributes (raw data) and psychometric scales BPRS and PANSS. Subjects indicated by name and surname initials. (TIF) [file pone.0034928.s006.tif]

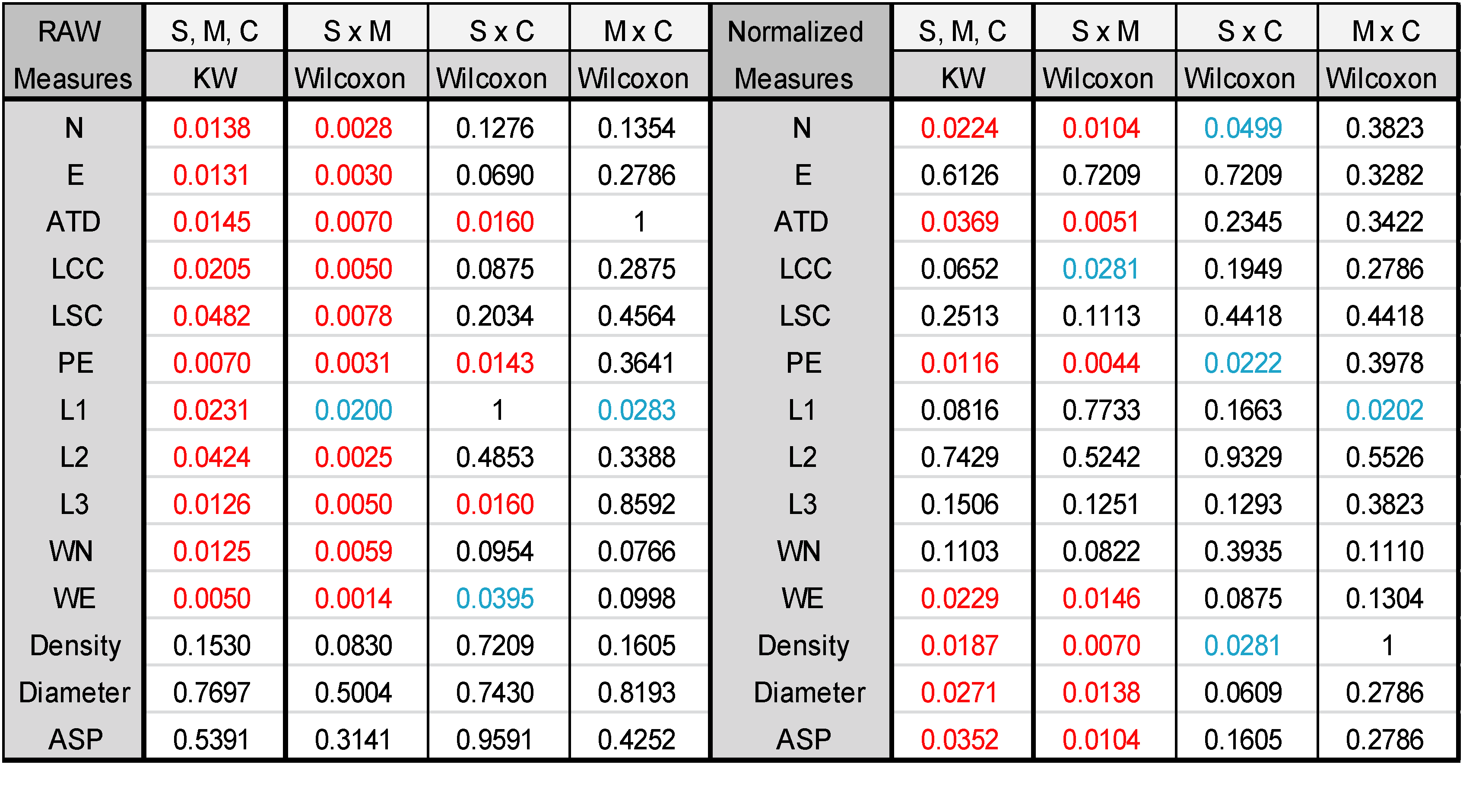

Supplement: Table S4 — P values obtained on the Kruskal-Wallis (KW) test followed by Wilcoxon-Ranksum test with Bonferroni correction for pairwise group comparisons of raw and normalized data for schizophrenics (S), manics (M) and controls (C). Statistically significant differences indicated in red, near-significant trends indicated in blue. (TIF) [file pone.0034928.s007.tif]

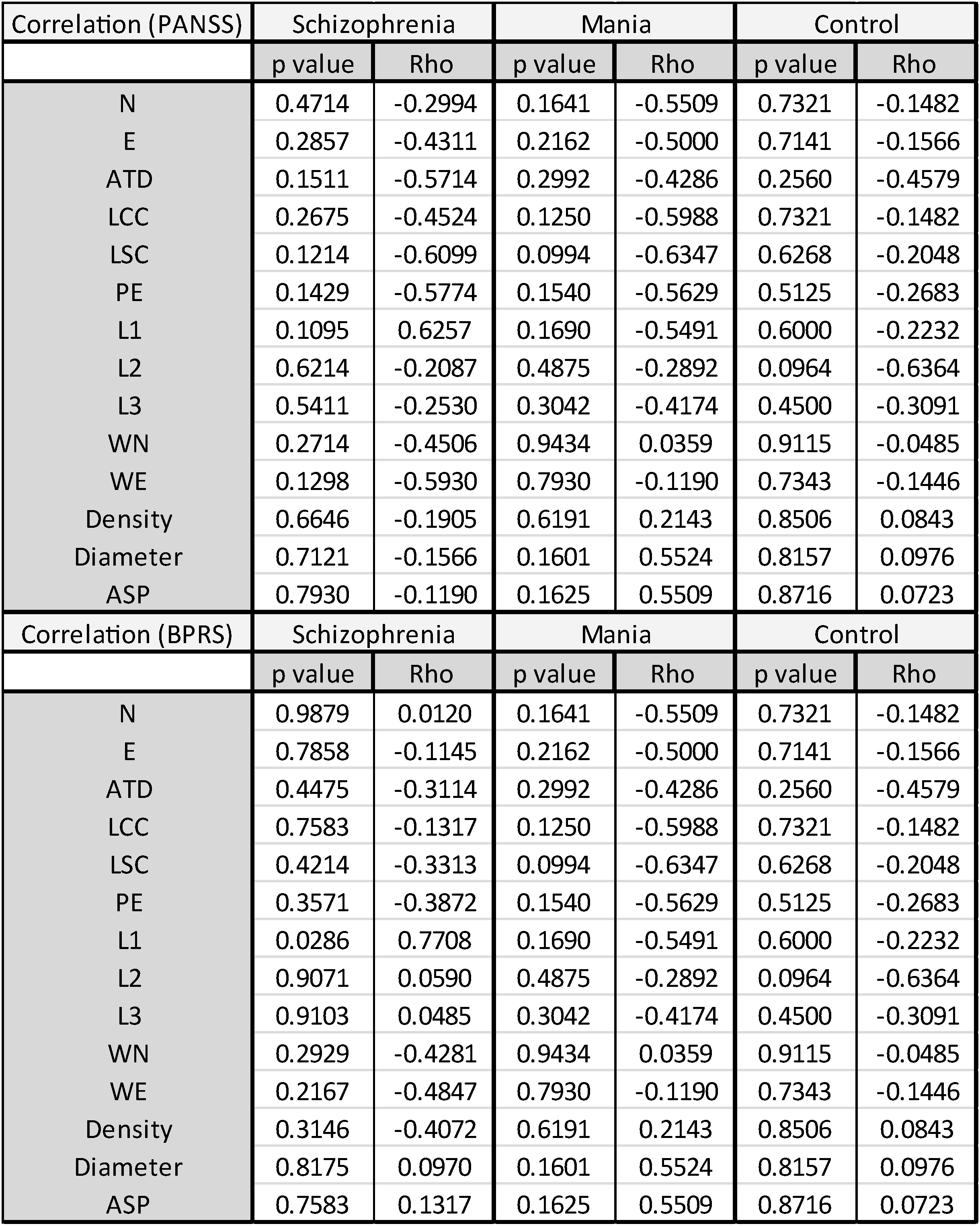

Supplement: Table S5 — There were no significant correlations between normalized graph attributes and psychometric scales (BPRS and PANSS scores). Shown are Rho and P values of Spearman correlations (corrected α = 0.0166). (TIF) [file pone.0034928.s008.tif]

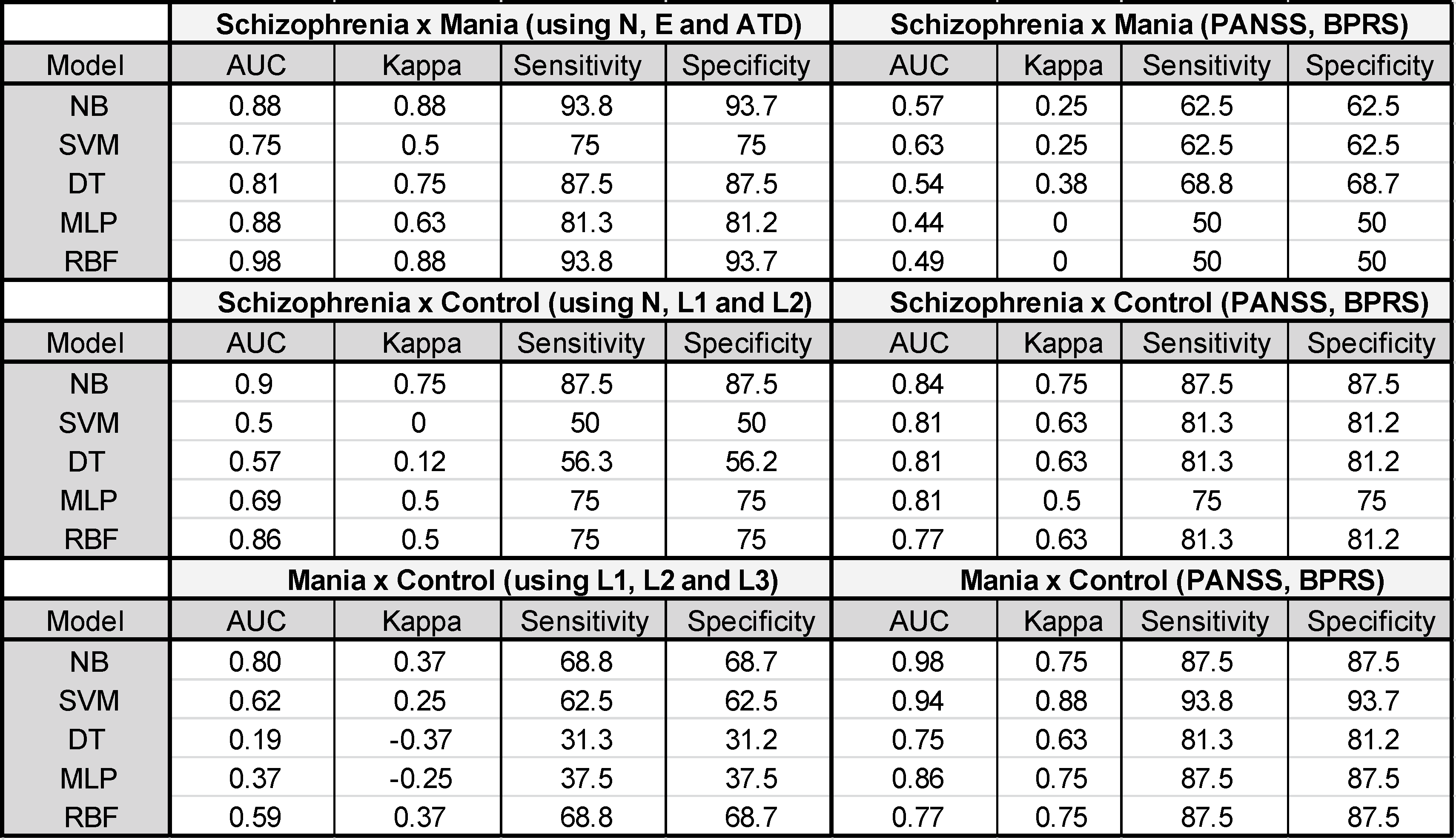

Supplement: Table S6 — Classification quality obtained for speech graph and psychometric measures. Five different binary classifiers were used: Naïve-Bayes (NB), Support Vector Machine (SVM), Decision Tree (DT), Multi-Layer Perceptron (MLP), and Radial Basis Function (RBF). (TIF) [file pone.0034928.s009.tif]
